# Highly Efficient Corrosion Inhibitor for Pure Iron and Aluminum Metals in Aggressive Acidic Medium: Experimental and Computational Study

**DOI:** 10.3390/ma19010114

**Published:** 2025-12-29

**Authors:** Aeshah H. Alamri

**Affiliations:** Chemistry Department, College of Science, Imam Abdulrahman Bin Faisal University, P.O. Box 76971, Dammam 31441, Saudi Arabia; ahalamri@iau.edu.sa

**Keywords:** corrosion inhibition, 5-Methyl-1H-benzotriazole, pure iron, pure aluminum, electrochemical studies, 3D profilometry analysis, DFT, molecular dynamics

## Abstract

**Highlights:**

**What are the main findings?**

- MHBTZ exhibits excellent corrosion inhibition performance for pure iron and aluminum in aggressive acidic media.
- EIS results reveal very high inhibition efficiencies of 98.94% for Fe and 99.16% for Al at 2500 ppm.
- PDP measurements confirm that MHBTZ acts as a mixed-type inhibitor, suppressing both anodic metal dissolution and cathodic hydrogen evolution.
- Experimental findings are strongly supported by DFT and MD simulations, indicating robust interactions between the inhibitor and metal.
- 3D optical profilometry demonstrates the formation of a compact and protective film on Fe and Al surfaces in the presence of MHBTZ.

**What are the implications of the main findings?**

- MHBTZ can be considered a highly effective organic inhibitor for protecting Fe- and Al-based materials in acidic industrial environments.
- The combined experimental–computational approach provides reliable mechanistic insight into corrosion inhibition behavior.
- The high efficiency at relatively low concentration highlights the potential of MHBTZ for cost-effective and practical corrosion control applications.

**Abstract:**

The influence of 5-Methyl-1H-benzotriazole (MHBTZ) on the corrosion of pure iron (Fe) and aluminum (Al) in 1 M HCl was investigated in this study. The experimental and theoretical aspects of MHBTZ adsorption onto pure iron (Fe) and aluminum metal (Al) surfaces, as well as the stability of adsorbed layers based on the metal type, were also studied. Different electrochemical measurements were performed to explore the corrosion rates and inhibition efficiencies on the Fe and Al surfaces at 298 K. Optical profilometry was used to obtain the 3D surface topography of Fe and Al metals after immersion with and without the MHBTZ molecule. The results showed that MHBTZ exhibited excellent inhibition properties for both metals. Electrochemical impedance spectroscopy (EIS) achieved inhibition efficiencies of 98.1% and 98.5% for Fe and Al, respectively, at a concentration of 2500 ppm. Potentiodynamic polarization (PDP) indicated that MHBTZ acted as a mixed-type inhibitor. Density functional theory (DFT) analysis and molecular dynamics (MD) simulations were used to explore the relationship between the molecular structure of MHBTZ and its inhibition efficiency at the atomic level.

## 1. Introduction

Corrosion is a major challenge faced by the industrial sector, as it affects all areas of operation, especially in environments such as chemical processing, desalination, and oil and gas production. Acid corrosion can cause significant material damage during critical operations, such as industrial cleaning and descaling processes [1,2,3]. The use of HCl and other acids for scale removal creates problems because they break down metal substrates, which damages equipment structures and shortens operational periods [4,5]. The worldwide economic damage caused by corrosion is approximately USD 2.5 trillion each year, highlighting the need for successful protective methods [6,7].

Corrosion inhibition is one of the solutions used in industry to minimize the corrosion rates of metals and alloys at a low cost. Organic compounds serve as fundamental components in corrosion inhibition science because their performance can be enhanced by modifying their molecular structures and physicochemical properties [8,9,10]. The mechanism of action of these inhibitors is adsorption on metal surfaces, thus creating a protective layer that blocks corrosive electrolyte access. The most effective corrosion inhibitors function through chemisorption, forming dense and stable films by establishing strong covalent or coordinate bonds with the metal surfaces. The effectiveness of organic inhibitors depends on the presence of particular functional groups, including heteroatoms (N, S, O, and P) and π-systems (aromatic or heterocyclic rings) that serve as adsorption sites [8,11,12,13,14,15].

In recent years, numerous studies have been conducted to develop organic inhibitors that can target metal species. For carbon steel, which mostly contains 99% pure iron, a vast body of research work has explored compounds that can mitigate its high susceptibility to acid-induced corrosion [4,16,17,18,19,20,21]. Similarly, for aluminum alloys that contain 98% pure Al, several studies have investigated different corrosion inhibitors ranging from amines and hydrazine derivatives to Schiff bases and surfactants [7,22,23,24,25,26,27,28].

Previous studies have extensively documented the performance of various triazole derivatives as corrosion inhibitors for carbon steel and aluminum in acidic media [29,30,31,32,33,34,35,36,37,38]. Research shows that specific derivatives reach up to 95% inhibition efficiency when evaluated against mild steel and aluminum alloys, but these experimental tests were conducted for individual metals with different sets of inhibitors [39,40,41,42,43,44,45].

In addition, the scientific literature indicates that information on the use of a corrosion inhibitor to protect multiple metals from corrosion during the acid cleaning process is scarce [38,46,47]. Therefore, this research is important because modern industrial operations require this capability as their production systems consist of assemblies that include multiple metal components.

This research investigates 5-Methyl-1H-benzotriazole (MHBTZ) as a dual-action molecule that demonstrates effective corrosion inhibition toward pure iron and aluminum in 1 M HCl solution. Electrochemical techniques combined with 3D optical profilometry analysis were employed to obtain inhibition efficiency and further confirm the adsorption of MHBTZ on the Fe and Al surfaces. Furthermore, DFT calculations at the GGA/PBE level and molecular dynamics (MD) simulations were used to provide further understanding of the inhibition effect of MHBTZ at the molecular level.

## 2. Experimental Section

### 2.1. Material and Solution

The preparation of the solutions required 5-Methyl-1H-benzotriazole (MHBTZ) and 1 Molar hydrochloric acid (1 M HCl). All reagents employed in this study were obtained from Sigma-Aldrich to ensure high analytical quality of the reagents. Test solutions were subsequently prepared at concentrations of 1000, 1500, 2000, and 2500 ppm MHBTZ solutions at room temperature (298 K). Corrosion experiments were conducted on the pure iron (Fe) and pure aluminum (Al) samples. Before each experiment, pure Fe and Al specimens were mechanically polished using successive grades of emery paper (400, 600, 800, and 1200 grit). Following the polishing procedure, the samples were thoroughly rinsed with distilled water to remove residual particles from the surface.

### 2.2. Electrochemical Measurements

Electrochemical studies were performed using a Gamry Interface 5000 E potentiostat/galvanostat (Gamry Instrument, Warminster, PA, USA). The working electrodes consisted of pure Fe and Al samples with a 0.5 cm^2^ exposed surface area. A saturated calomel electrode and graphite were used as the reference and the counter electrodes, respectively. The three-electrode system corrosion cell contained 150 mL of 1 M HCl solution, with and without the corrosion inhibitor. The experiments were first conducted for one hour at a steady state open-circuit potential (OCP) to ensure stability. Electrochemical impedance spectroscopy (EIS) analysis was subsequently performed with an AC signal with an amplitude of 10 mV at the OCP within a frequency range of 100 kHz to 0.1 Hz. This was followed by linear polarization resistance (LPR) measurements using a potential scan of ±0.1V relative to the OCP at a rate of 0.125 mV/s. Finally, potentiodynamic polarization (PDP) studies were obtained from −0.250 V to −0.25 V at OCP at a scan rate of 1.0 mV/s.

### 2.3. Computational Details

#### 2.3.1. Microspecies Analysis of Mhbtz

**Figure 1:**
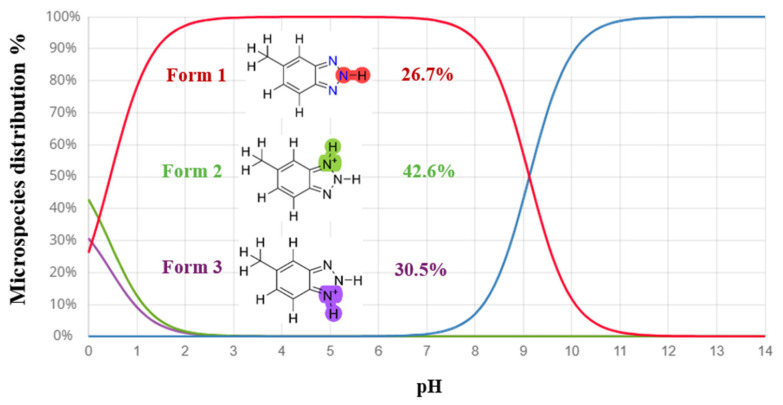
Distribution of MHBTZ forms as a function of pH.

The major microspecies of MHBTZ as a function of pH were identified using Marvin Sketch software (https://chemaxon.com/marvinsketch_js, accessed on 16 November 2025) within a pH range of 0.0 to 14.0. For the theoretical calculations, the microspecies present at pH = 0 were selected, because experimental investigations were carried out under acidic conditions (1 M HCl). The analysis revealed that MHBTZ exists as three distinct microspecies at pH = 0. The relative distribution was estimated to be 26.7% for the neutral form (Form 1), along with protonated species (Form 2 (42.6%) and Form 3 (30.5%)). Representative microspecies at pH = 0 are shown in Figure 1.

#### 2.3.2. Molecular Properties Calculations Using DFT Simulations

Density Functional Theory (DFT) calculations were performed using the Dmol^3^ module in Materials Studio 17 to optimize the molecular geometry of MHBTZ. The Perdew–Burke–Ernzerhof (PBE) functional within the generalized gradient approximation (GGA) was employed to describe the electronic exchange and correlation potential, together with all-electron calculations using a double-numeric basis set with polarization functions (DNP) [48,49]. Geometry optimizations were performed without imposing symmetry constraints, and vibrational frequency analyses were performed to verify that the optimized structure corresponded to the true minimum on the potential energy surface. To account for solvent effects, the COSMO continuum solution model was applied using water as the medium. All calculations were conducted using the fine convergence criteria [50]. Based on frontier molecular orbital theory, electronic parameters such as the highest occupied molecular orbital energy (E_HOMO_), lowest unoccupied molecular orbital energy (E_LUMO_), and energy gap (ΔE) were subsequently evaluated

(1) $$\Delta E=\;{\;E}_{LUMO\;}-\;E_{HOMO\;}$$

#### 2.3.3. MD Simulations

The Forcite module in Materials Studio was employed to investigate the interaction between the inhibitor molecules and the metallic surfaces of iron and aluminum [51]. Initially, the primitive abc unit cells of Fe and Al were optimized to their lowest-energy configurations, after which supercells of Fe (110) and Al (111) with dimensions of 10 × 10 were constructed, each consisting of five atomic layers derived from the optimized unit cells. Using the “build layers” tool, a simulation slab was generated by introducing a solvent layer containing water molecules together with the inhibitor species on the Fe (110) and Al (111) surfaces. The constructed box with periodic boundary conditions had a size of 24.82 Å × 24.82 Å × 57.61 Å for Fe (110) and 28.63 Å × 28.63 Å × 53.04 Å for Al (111). The MD simulation used the COMPASS II force field at 298 K under NVT ensemble conditions with a total simulation time of 100 ps and a 0.1 fs time step. During the simulations, Fe and Al atoms were constrained to their bulk positions, while one inhibitor molecule, nine Cl^−^ anions, nine H_3_O^+^ cations, and 491 H_2_O molecules were allowed to relax fully. The interaction energy (E_int_) between the inhibitor and Fe (110) and Al (111) surfaces was evaluated using the following expression [52]:

(2) $$E_{interaction}=\;E_{total}-(E_{surface}+\;E_{inhibitor})\;$$

### 2.4. Surface Analyses

#### 3D Profilometry Spectroscopy

The surfaces of the two metals, with and without MHBTZ, were characterized using a Profilm3D optical profiler (Filmetrics, San Diego, CA, USA) fitted with a Nikon 50× DI objective lens (Nikon Corporation, Minato, Japan), providing high-resolution three-dimensional imaging. The surfaces of Fe and Al metals were analyzed after immersion in a 1 M HCl solution at 25 °C with and without 2500 ppm for 6 h.

## 3. Results and Discussion

### 3.1. OCP Measurements

**Figure 2:**
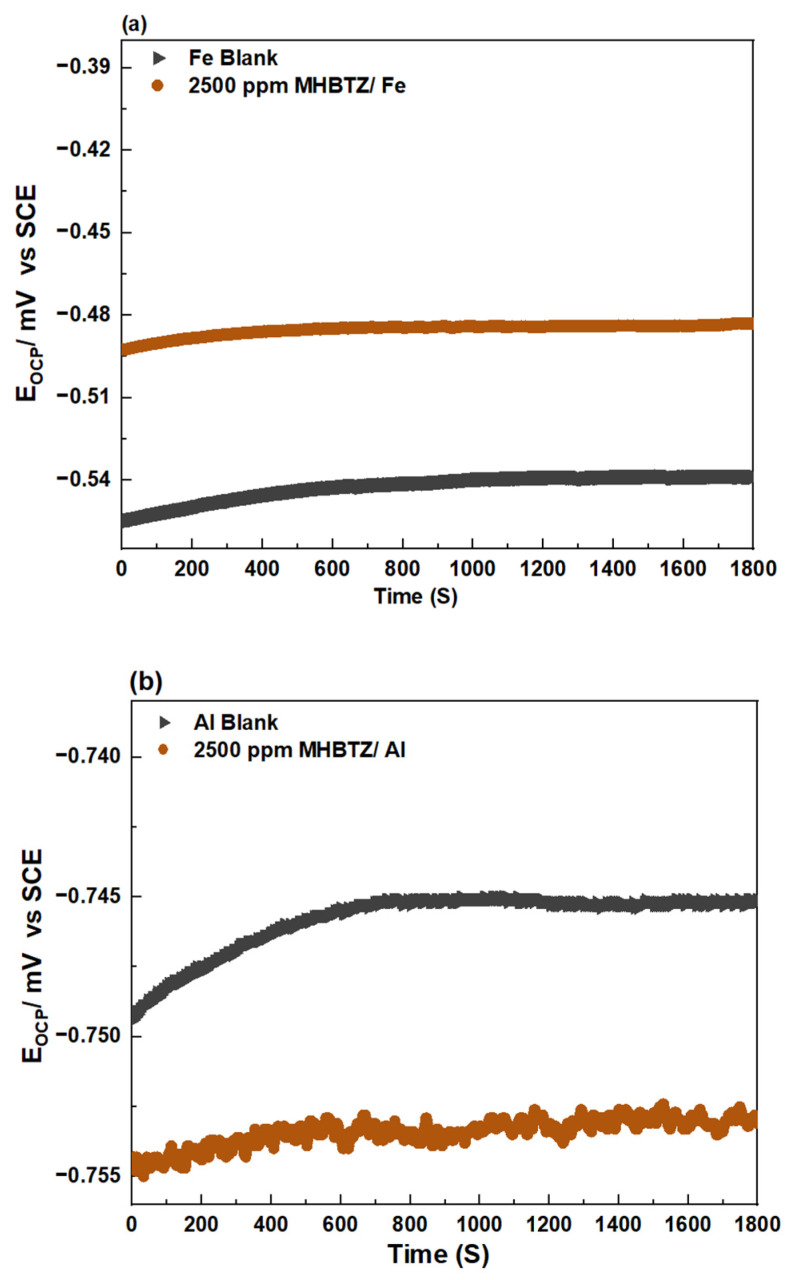
OCP variation of (**a**) Pure Fe, (**b**) Pure Al electrodes in 1 M HCl solution with and without the 2500 ppm MHBTZ at 25 °C.

Measurement of the open-circuit potential over time is a crucial technique that must be performed before each electrochemical test to achieve reliable results. The OCP versus time plots in Figure 2 show the behavior of the blank solution and the solutions containing 2500 ppm of MHBTZ (the concentration of 2500 ppm presented in this figure is a representative plot only, as concentrations ranging from 500–2500 ppm were investigated in this study). The OCP reached a stable state during the time used in this work (half an hour for pure Fe and Al metals). The OCP values were −0.52 V vs. SCE for Fe and −0.749 V vs. SCE for Al in the blank solution, indicating active corrosion of the pure Fe and Al substrates. The Al metal showed a strongly negative potential upon immersion in HCl solution because of its strong tendency to corrode in acid when compared to Fe. With the introduction of the optimum concentration of the inhibitor (2500 ppm MHBTZ), Al exhibited cathodic behavior relative to the blank system, indicating a more pronounced inhibitory effect on cathodic hydrogen production. The noisy OCP of Al metal in the presence of the inhibitor arises from the dynamic adsorption–desorption of inhibitor molecules, unstable oxide/inhibitor film formation, and localized activation events on the aluminum surface. In contrast, for Fe corrosion, the addition of 2500 ppm of MHBTZ shifted the potential toward positive values until it reached approximately −0.48 V. The positive shift in the corrosion potential confirmed the role of the inhibitor in suppressing anodic Fe corrosion through adsorption onto its surface [46,53].

### 3.2. EIS Measurement

**Figure 3:**
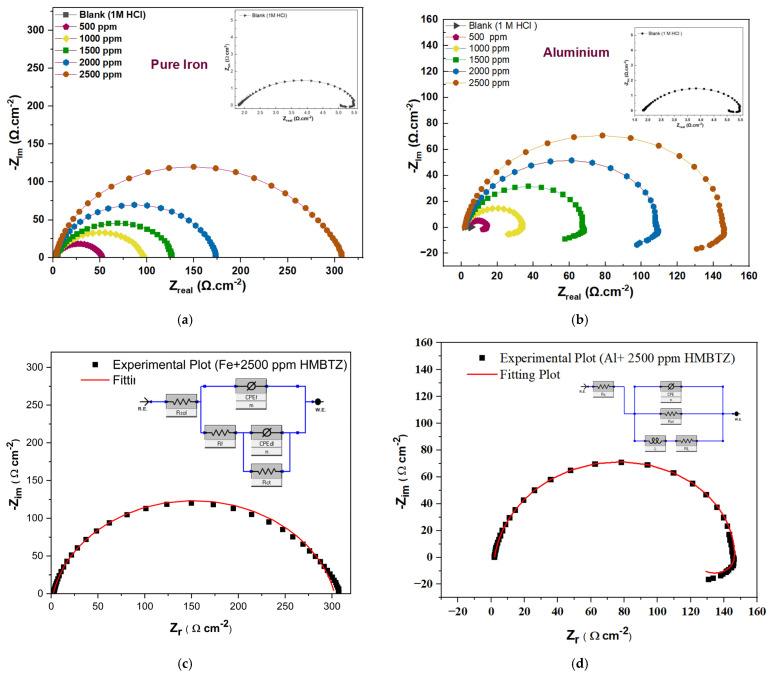
Nyquist plots of (**a**) Pure Fe, (**b**) Pure Al electrodes in 1 M HCl solution without and with different concentrations of MHBTZ at 25 °C. Representatives of an equivalent circuit suitable for EIS analysis of various metals of (**c**) pure iron and (**d**) aluminum at 2500 ppm.

The Nyquist plots in Figure 3 display single depressed semi-circles, which stem from the Fe surface heterogeneity due to impurities and surface roughness, together with dislocations and other factors [54]. To achieve reliable results and match the experimental data, the equivalent circuit uses a constant phase element (CPE) instead of a capacitor because it allows the model to consider a depressed semicircular shape. In this case, the capacitive loops expanded with increasing MHBTZ concentration, indicating better corrosion inhibition effects. In this case, the polarization resistance (R_p_) is the sum of the solution resistance (R_s_), the film resistance (R_f_) and charge transfer resistance (R_ct_).

R_p_ = R_s_ + R_f_ + R_ct_ (3)

The polarization resistance (R_p_) represents the combination of the film resistance (R_f_) and charge transfer resistance (R_ct_) because they were connected in series based on the equivalent circuit.

The Nyquist plots for aluminum displayed depressed semicircles that increased with increasing inhibitor concentration. However, aluminum showed inductive loops after capacitive loops, which led to the use of a different equivalent circuit for Al under the investigated conditions. The inductive behavior of aluminum in HCl is mainly attributed to the relaxation of adsorbed chloride-containing intermediates formed during oxide film breakdown and dissolution. It is also associated with localized depassivation, where coupled anodic dissolution and cathodic hydrogen evolution at corrosion fronts cause a delayed response. Additionally, time-dependent changes at the Al/solution interface can lead to an apparent low-frequency inductive loop in EIS spectra [55,56]. The polarization resistance (R_p_) was calculated using Equation (4).

(4) $$R_{p}\;=\;\frac{R_{L}\times \;R_{ct}}{{R_{L}\;+\;R}_{ct}}$$

The charge transfer (R_ct_) and inductive resistances (R_L_) were operated in parallel in the equivalent circuit [26]. The effective double-layer capacity (C_dl_) values obtained from the impedance data were calculated using the formula described in Equation (5) and presented in Table 1.

(5) $$C_{\mathrm{dl}}\;=\;{(Y}_{º}R_{p}^{1-n}{)}^{1/n}$$

R_p_ indicates the polarization resistance, Y_0_ measures the CPE coefficients (reciprocal impedance values also known as admittances), and n describes surface heterogeneity.

(6) $$IE\%=\;\frac{R_{p\;(inh)}-\;R_{p\;(blank)}}{R_{p\;(inh)}}\;\times 100$$

The inhibition efficiency (IE) (Equation (6)) was determined through polarization resistance (R_p_) measurements because this parameter serves as a standard method to evaluate electrochemical corrosion behavior. The R_p_(blank) value represents the resistance of the uninhibited solution, while R_p_(inh) shows the resistance values obtained with the inhibitor present. The difference between these two parameters directly indicates the extent to which the inhibitor protects the surface while minimizing the corrosion activity.

Table 1 lists the EIS parameters for iron and aluminum metals with and without the investigated inhibitor. The results showed that the double-layer capacitance (C_dl_) decreased when the MHBTZ concentration increased. However, R_p_ increased with increasing MHBTZ concentration and reached 302.78 Ωcm^2^ at the highest concentration. The highest R_p_ value corresponded to the maximum inhibition efficiency of MHBTZ, which reached 98.94% at 2500 ppm. The high R_p_ resistance was caused by the film resistance that appeared in the Fe equivalent circuit as the barrier film resistance at the electrode surface.

The electrochemical parameters from the aluminum results showed that the polarization resistance increases as the MHBTZ concentration increases, reaching its highest value of 201.67 Ω.cm^2^ at 2500 ppm. Enhanced resistance indicates shorter distances between the adsorbed inhibitor-metal surface, thus verifying the order of the inhibition performance while showing a decreased double-layer capacitance. The optimum concentration resulted in 99.16% inhibition. Moreover, the charge transfer resistance (R_ct_) and induction resistance (R_L_) increased when the MHBTZ concentration increased, whereas the double layer capacitance decreased until it reached 27.95 μF·cm^−2^ at 2500 ppm. The equivalent circuit model for the aluminum working electrodes includes an inductor, which indicates the presence of induction resistance. The equivalent circuit model for Fe metal does not include this parameter because it lacks an inductor element.

MHBTZ exhibited excellent inhibitory properties under the examined conditions by forming an effective protective barrier. The protective barrier on the Fe and Al metal surfaces resulted from the adsorption of MHBTZ molecules. This adsorption behavior arises from the benzotriazole and phenyl rings in the molecular structure. The experimental results demonstrate that MHBTZ exhibits strong reactivity, which is consistent with theoretical predictions.

EIS was employed to evaluate the performance of the MHBTZ molecules against pure Fe and Al metals in 1 M HCl at 298 K, both with and without MHBTZ. Figure 3 shows Nyquist plots and the equivalent circuit simulations for each metal.

### 3.3. LPR Measurement

The efficiency of MHBTZ as a corrosion inhibitor in 1 M HCl was assessed using the linear polarization resistance (LPR) method. This electrochemical method provides a reliable, non-destructive, and real-time evaluation of corrosion rates and is widely employed to quantify the protective performance of inhibitors. In this approach, a small potential perturbation of ±10 mV relative to the OCP was applied, establishing a direct relationship between the corrosion current density i_corr_ and the measured potential response. The polarization resistance Rp was determined using Equation (7) [57], where βa and βc are the anodic and cathodic Tafel slopes, respectively.

(7) $$R_{p}=\;\frac{\beta_{c}\;\times \beta_{a}}{2.3\;\left(\;\beta_{c}\;+\beta_{a}\right)i_{corr\;}}\;$$

The inhibition efficiency (IE%) was subsequently calculated using Equation (8):

(8) $$IE\%=\;\frac{R_{p\;(inh)}-\;R_{p\;(blank)}}{R_{p\;(inh)}}\;\times \;100$$

Rp (blank) denotes the polarization resistance of the uninhibited solution, whereas Rp (inh) corresponds to the values obtained in the presence of the inhibitor. These parameters provide quantitative insights into the ability of the tested compounds to suppress both anodic and cathodic processes during the corrosion of pure iron and pure aluminum (±standard deviation), as shown in Table 2.

### 3.4. PDP Measurement

**Figure 4:**
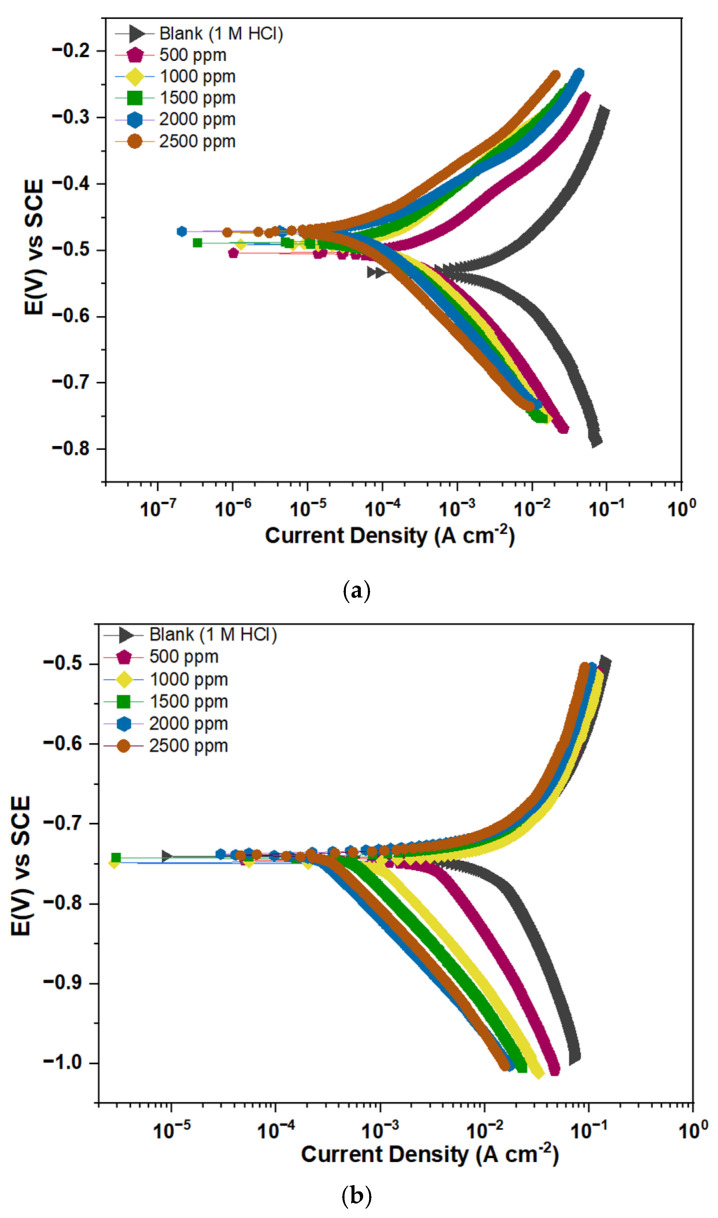
Polarization curves of (**a**) Pure Fe, (**b**) Pure Al electrodes in 1 M HCl solution without and with different concentrations of MHBTZ at 25 °C.

To understand the corrosion kinetics at the Fe/Al metal solution interfaces, PDP measurements were performed. The recorded and displayed polarization curves for Fe and Al immersed in 1 M HCl with and without MHBTZ are shown in Figure 4. The electrochemical parameters (i_corr_, E_corr_, ß_c_, and ß_a_) were obtained by extrapolating both the anodic and cathodic regions using the Gamry Echem Analyst program and are presented in Table 3. The addition of the MHBTZ inhibitor caused a shift in the obtained plots toward lower corrosion current densities compared with the uninhibited solution, based on the polarization curves. The reduction in i_corr_ values became more pronounced with increasing inhibitor concentrations. The inhibitor demonstrated dual functionality, blocking both the anodic and cathodic reactions. MHBTZ was absorbed onto Fe and Al metal surfaces by slowing down hydrogen evolution reactions and preventing chlorides from contacting the electrode surface [24,58].

The Polarization curves of (Figure 4a) Pure Fe and (Figure 4b) Pure Al electrodes in 1 M HCl solution without and with different concentrations of MHBTZ at 25 °C are presented in Figure 4. The result shows that MHBTZ slows down hydrogen evolution more effectively than metal dissolution for both pure Fe and Al metals, as evident in their distinct parallel cathodic curves [47]. The i_corr_ values for Fe metal decreased as the MHBTZ concentration increased until it reached 0.103 mA·cm^−2^, resulting in 99.20% inhibition at 2500 ppm. On the other hand, the Al metal achieved an inhibition level of 99.27% at the same concentration, with a corrosion current density of 0.703 mA·cm^−2^.

The cathodic Tafel slope (β_c_) did not exhibit significant variations with increasing inhibitor concentration, indicating that hydrogen evolution followed a pure activation process [59,60]. The MHBTZ inhibitor protects both the anodic and cathodic processes at the Fe and Al metal surfaces through its mixed inhibition properties. The polarization curve results shown in Figure 4 confirm this finding [61,62].

The high inhibition level, which produces minimal corrosion current densities, can be attributed to the enhanced surface coverage by adsorbed molecules on the electrode surface. The formation of a barrier film is improved, which protects the material from aggressive environments [63]. The PDP analysis results followed the order of inhibition performance determined by LPR measurement.

The inhibition efficiencies were calculated from the i_corr_ values using Equation (9):

(9) $$IE\%=\;\frac{i_{corr\;(blank)}-\;i_{corr\;(inh)}}{i_{corr\;(blank)}}\;\times 100$$

The corrosion current densities of the sample in 1 M HCl solution with and without inhibitors are denoted by i_corr_ (blank) and i_corr_ (inh), respectively.

### 3.5. Computational Details

#### 3.5.1. Chemical Reactivity Prediction of MHBTZ

Quantum chemical calculations have become powerful tools for predicting molecular reactivity before experimental validation, thereby minimizing unnecessary effort, costs, and time. In this study, the DMol^3^ module executes all the quantum chemical calculations using DFT methods. Density Functional Theory (DFT) calculations were performed at the PBE/GGA/DNP level to gain insights into the structural and electronic properties of MHBTZ and evaluate its potential as a corrosion inhibitor. Because the central aim of this study was to assess the applicability of MHBTZ in acidic environments, particular attention was given to the species expected to dominate under such conditions. The possible protonated forms were identified using the Marvin Sketch software to approximate the experimental environment. As illustrated in Figure 1, the second protonated form (Form 2) was predicted to be the most stable species at highly acidic pH, exhibiting the highest distribution percentage near pH 0.

**Figure 5:**
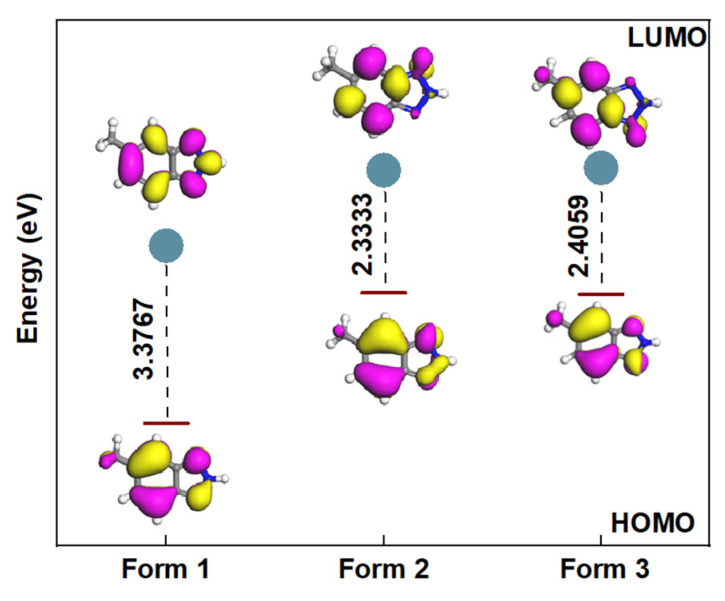
HOMO, LUMO and energy gaps (in eV) of MHBTZ forms using in their abundant forms at pH = 0 in aqueous solution.

Aqueous phase optimization of the MHBTZ molecule resulted in a complete geometric configuration determination, as shown in Figure 5. The chemical activity of the molecule was determined by analyzing the two frontier molecular orbitals, LUMO and HOMO. The E_LUMO_ shows the ability of a molecule to accept electrons, whereas E_HOMO_ shows the potential of a molecule to donate electrons. The energy gap (ΔE) between the molecular orbitals is equal to E_LUMO_-E_HOMO_. Figure 5 shows that Form 2 exhibits the lowest energy gap. This implies that Form 2 is the most reactive and has the highest potency for interacting with the Fe and Al surfaces compared to Forms 1 and 3.

**Figure 6:**
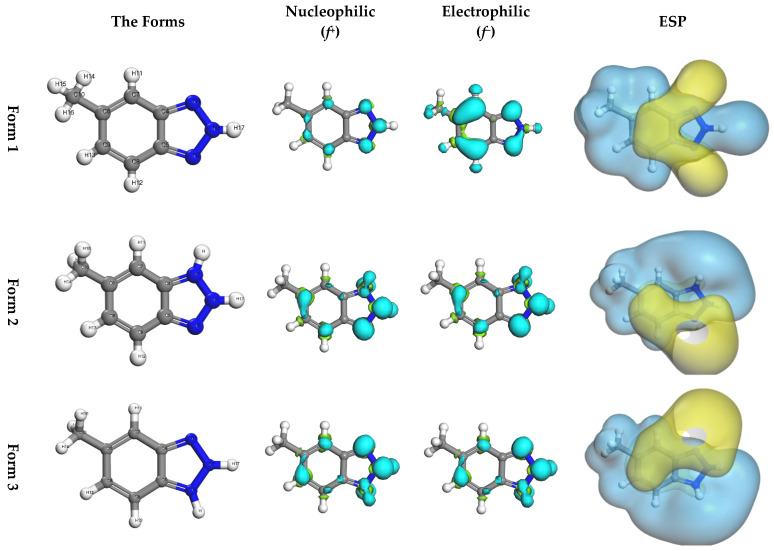
Optimized geometries, nucleophilic (*f*^+^), electrophilic (*f*^−^) and molecular electrostatic potential (ESP) of the three MHBTZ forms.

**Figure 7:**
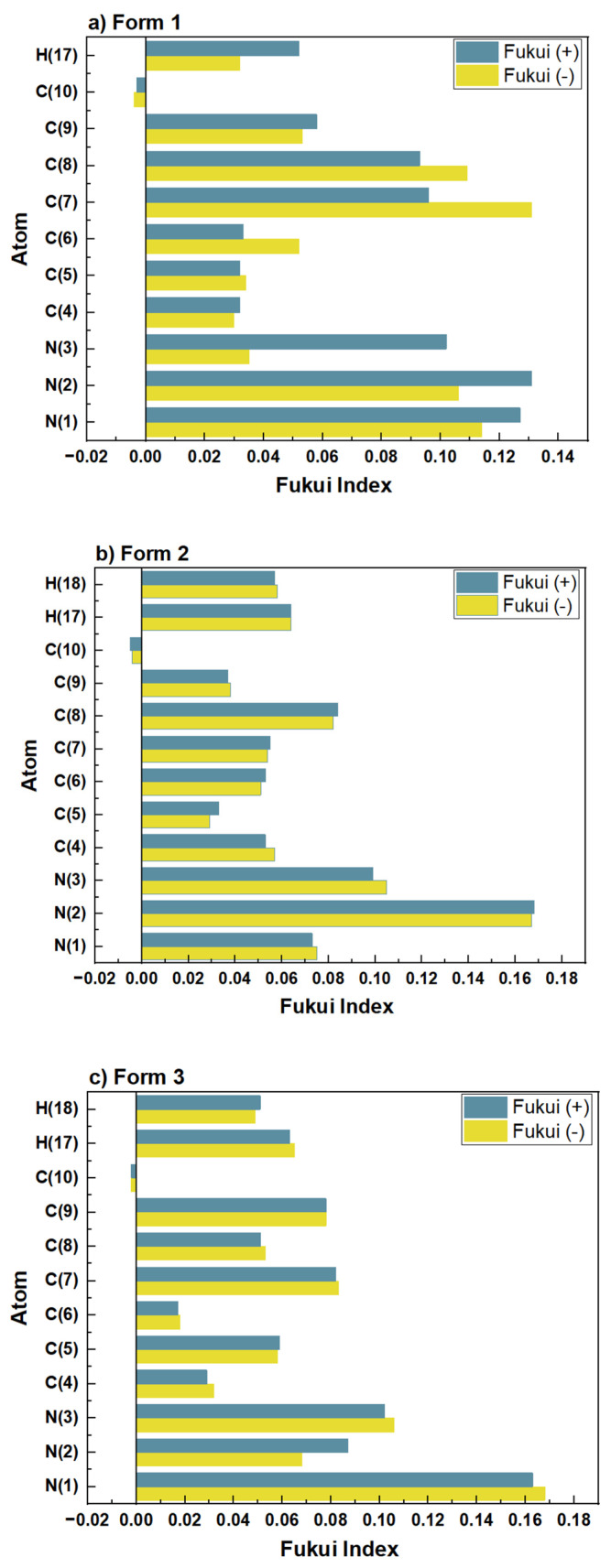
Fukui functions for nucleophilic and electrophilic sites of the three MHBTZ forms.

Furthermore, the Fukui function provides atomic-level insights into the potential electron transfer pathways between MHBTZ molecules and the surfaces of iron and aluminum. This approach highlights the specific reactive centers responsible for adsorption, thereby strengthening the mechanistic understanding of the inhibition process. The nucleophilic and electrophilic properties of MHBTZ are shown in Figure 6 and Figure 7, respectively. The Fukui indices (ƒ^+^ and ƒ^−^) were calculated using Mulliken population analysis to perform a detailed assessment of the local reactivity. The Fukui indices show which atoms in the MHBTZ molecules accept or donate electrons during nucleophilic, electrophilic, or radical attacks. For a molecular structure, the nucleophilic Fukui function (ƒ^−^) indicates locations prone to providing electrons, whereas the electrophilic Fukui function (ƒ^+^) indicates regions that are inclined to accept electrons. For instance, higher ƒ^+^ values were concentrated at the N1, N2, and N3 atoms in Form 1–3. Similarly elevated ƒ^−^ values were prominent at the C7, C8, N1 and N2 atoms in all forms. Notably, these findings from the Fukui indices shed light on the reaction sites between donors and recipients for all forms of MHBTZ, thus identifying the metal surface adsorption sites at the atomic level.

#### 3.5.2. Molecular Dynamics (MD) Simulations

**Figure 8:**
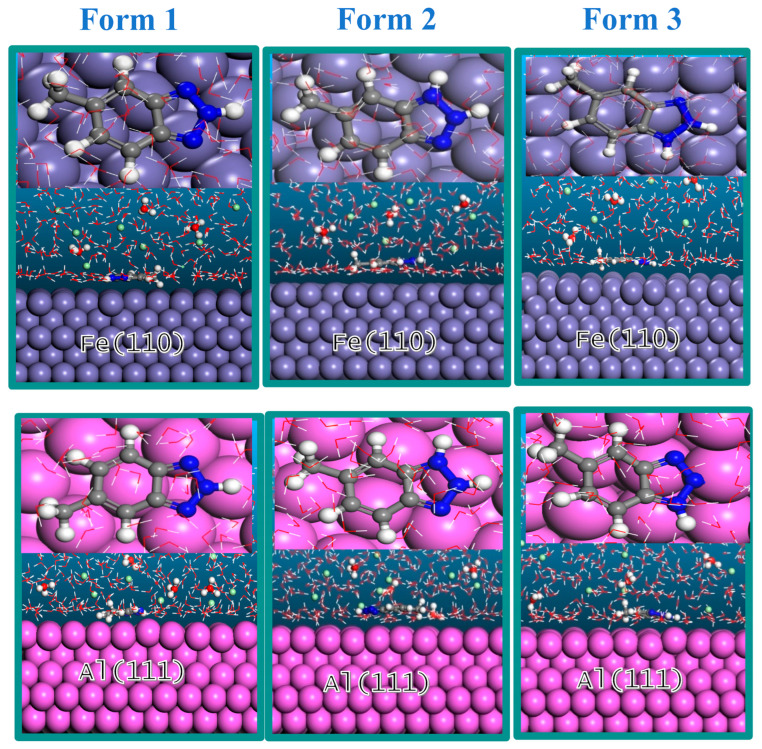
Most stable adsorption configurations of the three forms of the MHBTZ inhibitor on Fe (110) and Al (111) surfaces. Gray (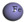) =Fe, Purple (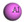) = Al, Dark gray (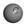) = C, Green (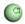) = Cl, Red (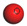) = O, Blue (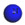) = N, White (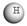) = H.

Molecular dynamics (MD) simulations were used to obtain the interaction energies between the Fe and Al metal surfaces and the three different forms of MHBTZ. Before conducting the MD simulation, the system was equilibrated to achieve a stable temperature and energy. The most stable adsorption positions of MHBTZ forms on the Fe and Al metal specimens are shown in Figure 8. The adsorption sites that drive the adsorption process mainly consist of N atoms located on the triazole and charge distribution on the phenyl ring structure found in the inhibitor molecule. The MHBTZ molecule aligns parallel to the metal surface, resulting in complete surface coverage and solidification of the strong interaction bond between the adsorbate and substrate systems. Table 4 presents the interaction energies of the three forms of the MHBTZ inhibitor on Fe (110) and Al (111) surfaces. The energy values presented in Table 4 were calculated according to Equation (2), which defines the interaction energy of the inhibitor-metal system. The interaction energy of form 2 of MHBTZ was the most negative for the Fe (110) surface (−103.53 kcal/mol) and Al (111) surface (−65.61 kcal/mol). The high negative energy interaction indicates a stronger interaction between Form 2 of MHBTZ and the two metal surfaces. This indicates that among the different forms of MHBTZ, Form 2 is the most important in acidic solutions.

### 3.6. Optical Profilometry Analysis

**Figure 9:**
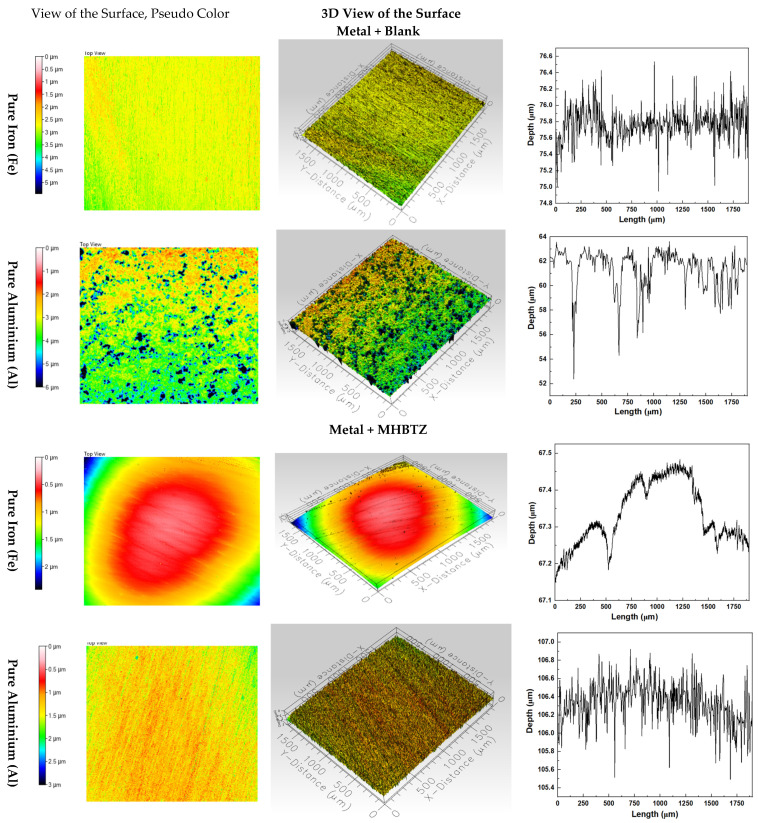
Three-dimensional morphology scanning results for the front side of the pure metals after 4 h immersion in 1 M HCl solution (with and without 2500 ppm of MHBTZ as corrosion inhibitor).

The 3D optical profilometer is a vital instrument for studying pitting corrosion and metal surface roughness under both corrosion inhibitor-free and inhibitor-present conditions [64,65]. The optical profilometry (2D and 3D) images in Figure 9 demonstrate the surface conditions of pure Fe and Al metals after 4 h in 1 M HCl solution with and without 2500 ppm MHBTZ corrosion inhibitor. The addition of corrosion resulted in substantial surface quality enhancement of iron and aluminum materials (±standard deviation), according to the 3D profilometry results shown in Figure 9 and Table 5, respectively. The iron surface experienced a substantial reduction in the Arithmetic Mean Height (Sa) value from 0.26 µm to 0.16 µm because of the ability of the inhibitor to fight general corrosion. The inhibitor had an enhanced effect on aluminum surfaces, as the Sa value decreased from 0.80 µm to 0.18 µm, indicating a substantial improvement in surface smoothness. Sq is represented by the Root Mean Square Height, which shows the root mean square value of surface height measurements relative to the mean plane, making it suitable for surface roughness evaluation. The surface appeared more even when the Sq values were low. The value of the iron and aluminum surface decreased from 0.34 in the blank solution to 0.20 in the inhibited solution for Fe, and from 1.16 to 0.23 for Al, which measures the effectiveness of corrosion inhibitors in surface protection. Moreover, the Ssk parameter indicates how the surface height data points are distributed across the measurement area. The presence of deep pits or valleys on the surfaces. The application of inhibitors resulted in changes in the Ssk value, indicating both reduced pit depth and frequency, as well as a surface transition from pitting to uniform corrosion. The Ssk value of iron and aluminum decreased from −0.87 in the blank solution to −0.17 in the inhibited solution for Fe, and also from −2.14 to −0.38 for Al metal. The kurtosis (Sku) value of the aluminum surface decreased dramatically from 10.82 to 3.95, indicating that the inhibitor transformed severe localized pitting corrosion into a more uniform and less aggressive corrosion pattern. The formation of the protective layer was confirmed by a substantial decrease in the Maximum Peak Height (Sp), Maximum Valley Depth (Sv), and maximum peak-to-valley height (St). values for both metals, demonstrating the ability of the inhibitor to minimize the surface deterioration. The inhibitor was exceptionally effective in blocking localized corrosion on aluminum surfaces, which is essential for maintaining material durability in harsh environments [66].

## 4. Conclusions

This study was motivated by the need to encourage the development of multi-purpose corrosion inhibitor systems that offer sufficiently high efficiency against acid cleaning-induced corrosion of multi-metallic industrial equipment. This minimizes expenses and encourages convenience for the industry. In this study, 5-Methyl-1H-benzotriazole (MHBTZ) was investigated for its ability to mitigate acid corrosion of Fe and Al metals. In this study, experimental electrochemical techniques and theoretical approaches were employed to assess the inhibition performance of MHBTZ on iron and aluminum in 1 M HCl. This combined strategy offers valuable insights into protective mechanisms by linking molecular reactivity with electrochemical behavior. The following conclusions were obtained from this study:

1. MHBTZ acts as a good corrosion inhibitor for pure Fe and Al in 1 M HCl, confirming the high reactivity of this derivative. It achieved inhibition percentages of 97.82% and 96.09% for Fe and Al pure metals, respectively.
2. MHBTZ was adsorbed on Fe and Al metal surfaces using mostly N atoms in its triazole ring. These sites are also the centers of protonation through which MHBTZ engages in the respective adsorption and inhibition mechanisms for the two pure metals.
3. For Fe corrosion in HCl, the polarization curves revealed that MHBTZ acted as a mixed-type inhibitor, inhibiting both the metal dissolution/hydrogen evolution reaction. However, for Al corrosion, the polarization curves show mainly cathodic inhibition, indicating a hydrogen evolution mechanism.
4. The 3D surface optical profilometer analysis confirmed the adsorption of the MHBTZ inhibitor on the pure Fe and Al metal surfaces and the reduction in localized corrosion.
5. A DFT study based on the frontier molecular orbital theory revealed that the MHBTZ molecule can exhibit good reactivity because it has various active sites that act as nucleophiles and/or electrophiles.
6. MD simulations revealed that the MHBTZ molecule exhibited a high negative interaction energy on the Fe and Al metal surfaces, indicating a strong interaction and, by extension, the high inhibition efficiencies obtained experimentally.

## Figures and Tables

**Table 1:** EIS parameters for pure Fe and pure Al after immersion in 1M HCl solution with (without) corrosion inhibitor (2500 ppm MHBTZ) at 25 °C.

Metal	Con. (ppm)	R_S_ (Ωcm^2^)	R_Ct_ (Ωcm^2^)	CPE_dl_	n	CPE_f_	m	R_f_ (Ωcm^2^)	C_dl_ μF·cm^−2^	Rp (Ωcm^2^)	IE %
				Y_01_		Y_02_					
				(µΩ^−1^s^n^cm^−2^)		(µΩ^−1^s^m^cm^−2^)					
**Fe**	0	1.5	0.83	445.72	0.93	253.71	0.95	0.88	770.15	5.65	-
	500	1.89	1.54	169.41	0.97	260.70	0.81	60.78	225.83	52.58	95.00
	1000	2.01	5.03	140.62	0.94	235.01	0.78	90.69	196.93	97.73	96.72
	1500	1.95	12.51	113.92	0.95	133.92	0.84	171.51	192.00	185.97	98.27
	2000	1.77	6.64	87.83	0.95	92.04	0.86	183.7	1.06	180.11	98.34
	2500	2.19	4.68	65.14	0.99	71.36	0.86	296.91	1.07	302.78	98.94
**Al**		**R_S_** **(Ωcm^2^)**	**R_Ct_** **(Ωcm^2^)**	**CPE_dl_** **(µΩ^−1^s^n^cm^−2^)**	**n**		**L**	**R_L_**	**C_dl_** **μF·cm^−2^**	**Rp** **(Ωcm^2^)**	**IE** **%**
	0	1.74	3.69	187.6	0.89	-	2.40	3.14	382.54	1.70	-
	500	1.84	45.09	155.2	0.81	-	0.07	16.50	307.61	12.08	85.96
	1000	1.98	53.75	32.4	0.90	-	0.11	72.15	69.73	30.56	94.45
	1500	2.35	174.71	24.93	0.92	-	0.16	102.01	47.37	64.41	97.37
	2000	2.5	179.91	21.38	0.93	-	0.25	174.61	37.73	88.61	98.09
	2500	2.04	271.22	18.15	0.95	-	0.79	786.50	27.95	201.67	99.16

**Table 2:** LPR results for pure Fe and pure Al after immersion in 1M HCl solution with (without) corrosion inhibitor (2500 ppm MHBTZ) at 25 °C.

Compounds	Con.	E_corr_	i_corr_	Rp	CR	IE
MHBTZ	(ppm)	(mV)	(mA/cm^2^)	(Ωcm^2^)	(mpy)	%
**Fe**	0	−538.45 ± 0.636	3.625 ± 0.417	6.995 ± 0.502	3583 ± 2.828	-
	500	−518.9 ± 0.141	0.550 ± 0.070	50.53 ± 2.234	457.95 ± 1.344	86.12 ± 1.607
	1000	−504.15 ± 0.212	0.265 ± 0.070	97.675 ± 1.351	245.45 ± 0.778	92.84 ± 0.415
	1500	−501 ± 0.0	0.215 ± 0.070	123.33 ± 4.497	189.5 ± 2.121	94.32 ± 0.614
	2000	−483.85 ± 0.212	0.155 ± 0.070	178.135 ± 2.708	134 ± 1.414	96.07 ± 0.222
	2500	−483.3 ± 0.424	0.085 ± 0.070	307.32 ± 3.083	77.67 ± 0.467	97.72 ± 0.141
**Al**	0	−744.1 ± 0.141	24.685 ± 0.233	1.06 ± 0.014	10630 ± 56.569	-
	500	−755.95 ± 0.070	8.37 ± 0.438	3.13 ± 0.141	3660 ± 91.924	66.12 ± 1.079
	1000	−758.050 ± 0.070	6.95 ± 0.480	3.76 ± 0.269	2949 ± 76.368	71.72 ± 2.397
	1500	−753 ± 0.070	2.285 ± 0.007	11.405 ± 0.021	991 ± 16.971	90.71 ± 0.107
	2000	−750 ± 0.0	1.075 ± 0.049	24.28 ± 1.061	452.75 ± 31.466	95.63 ± 0.249
	2500	−750 ± 0.212	0.925 ± 0.035	28.615 ±1.761	391.1 ± 23.900	96.29 ± 0.278

**Table 3:** PDP results for pure Fe and pure Al after immersion in 1M HCl solution with (without) corrosion inhibitor (2500 ppm MHBTZ) at 25 °C.

Compounds MHBTZ	Con. (ppm)	E_corr_ (mV)	i_corr_ (mA/cm^2^)	βa (mV/Decade)	Βc (mV/Decade)	CR (mpy)	IE %
**Fe**	0	−533	12.90	181	185	5910	-
	500	−504	0.709	122	121	324	94.52
	1000	−491	0.381	100	119	174	97.06
	1500	−488	0.230	92	112	105	98.22
	2000	−471	0.166	78	109	75	98.73
	2500	−472	0.103	68	108	47	99.20
**Al**	0	−740	96.501	232	576	41,410	
	500	−745	12.403	63	412	5344	87.09
	1000	−749	2.660	32	192	1143	97.24
	1500	−742	1.260	21	188	540	98.70
	2000	−739	0.756	21	160	324	99.22
	2500	−738	0.703	19	152	301	99.27

**Table 4:** Interaction energy of the three forms of the MHBTZ inhibitor on Fe (110) and Al (111) surfaces (Kcal/mol).

System MHBTZ	Total	Surface	Inh	Interaction Energy
**Form 1/Fe**	−6045.72	−5981.19	32.94	−97.47
**Form 2/Fe**	−6356.98	−6299.85	46.40	−103.53
**Form 3/Fe**	−6049.24	−5983.81	35.90	−101.33
**Form 1/Al**	−5754.36	−5719.67	19.95	−54.64
**Form 2/Al**	−5684.03	−5660.02	41.59	−65.61
**Form 3/Al**	−11670.56	−11648.95	37.91	−59.52

**Table 5:** Summarizes the 3D profilometry parameters of pure metals after immersion in electrolytes with (without) corrosion inhibitor (2500 ppm MHBTZ) after 4 h.

Sample ID	Sa (µm)	Sq (µm)	Ssk (µm)	Sku (µm)	Sp (µm)	Sv (µm)	St (µm)
**Metal + Blank**							
Fe	0.26 ± 0.028	0.34 ± 0.035	−0.87 ± 0.077	5.70 ± 0.658	3.16 ± 0.523	2.83 ± 0.226	5.99 ± 0.750
Al	0.80 ± 0.078	1.16 ± 0.120	−2.14 ± 0.205	10.82 ± 0.714	3.69 ± 0.028	13.49 ± 1.626	17.18 ± 1.655
**Metal + MHBTZ**							
Fe	0.16 ± 0.007	0.20 ± 0.0	−0.17 ± 0.099	3.52 ± 0.262	0.79 ±0.156	1.35 ± 0.262	2.14 ± 0.417
Al	0.18 ± 0.00	0.23 ± 0.0	−0.38 ± 0.0	3.95 ± 0.0	1.32 ± 0.0	1.68 ± 0.007	3.00 ± 0.007

## Data Availability

The original contributions presented in this study are included in the article. Further inquiries can be directed to the corresponding author.
